# Coupling coordination development of energy-economy-carbon emissions in China under the background of “double carbon”

**DOI:** 10.1371/journal.pone.0277828

**Published:** 2022-12-05

**Authors:** Zhiyuan Dong, Zenglian Zhang, Fengyuan Zhang

**Affiliations:** 1 School of Economic and Management, University of Science and Technology Beijing, Beijing, China; 2 School of Environment and Natural Resources, Renmin University of China, Beijing, China; Huanggang Normal University, CHINA

## Abstract

Based on the panel data of 30 provinces in China from 2010 to 2019, this paper measured the coupling coordination development of energy-economy-carbon emissions and investigated its regional differences and spatial convergence. The research methods in this paper include entropy weight technique method for order preference by similarity to an ideal solution, coupling coordination degree model, Dagum Gini coefficient and decomposition method, Moran’s I index, σ convergence model and β convergence model. The study found that the coupling coordination degree of energy-economy-carbon emissions in China has been continuously improved and has obvious regional and stage characteristics, but it is still on the verge of imminent disorder; the overall difference in the coupling coordination degree of energy-economy-carbon emissions shows a decreasing and then increasing trend, the main source of which is inter-regional differences; the coupling coordination degree of energy-economy-carbon emissions has a positive spatial correlation; except for the Southern Coastal Economic Zone and the Middle Yangtze River Economic Zone, there is no significant σ-convergence and β-convergence in the coupling coordination degree of energy-economy-carbon emissions system in other economic zones; the coupling coordination degree of energy-economy-carbon emissions changes fastest in the Middle Yangtze River Economic Zone. The innovation of this paper is to measure the coupling coordination degree of energy-economy-carbon emissions and to analyse its regional differences and spatial effects. It is of great practical significance to promote the coupling coordination development and regional balanced development of energy-economy-carbon emissions in China under the background of "dual carbon".

## 1. Introduction

Since the reform and opening up, China has made world-renowned achievements in economic development, and the total economic output and living standards of the people have continued to rise. Along with the continuous development of the economy, energy and environmental problems such as the rapid growth of energy consumption and the continuous rise in greenhouse gas and pollution emissions have been coming along. According to data from the International Energy Agency and the BP Statistical Yearbook of World Energy, China is the largest carbon dioxide emitter in world, accounting for 32% of the total carbon emissions in 2020. Under the dual pressure of energy and the environment, at the general debate of the 75th UN General Assembly in September 2020, General Secretary Xi Jinping clearly stated that China will adopt more powerful policies and measures to reach peak carbon dioxide emissions before 2030, striving to achieve carbon neutrality by 2060. In March 2021, General Secretary Xi Jinping reemphasized the need to integrate "Carbon Neutrality" into the overall layout of ecological civilization. From a practical point of view, various data clearly show that there is a strong link between the economy, energy and carbon emissions [1–3]. Under the background of "double carbon", it is important to accurately measure the coupling coordination of energy, economy and carbon emissions, reduce regional differences and realise spatial correlation, in order to realise the positive interaction and synergistic development of energy, economy and carbon emissions, and ultimately promote energy conservation and emission reduction and green low-carbon economic development in China.

In recent years, a great deal of research has been carried out on energy, economy and carbon emissions, which can be broadly divided into three categories. First, research on the relationship between economic growth and energy consumption. Some scholars [4–6] used Granger causality test to investigate the relationship between economic growth and energy consumption using time series data, and the study found that there is a unidirectional causality from economic growth to energy consumption. Using data from South Asian countries from 1975–2010 as a research sample, Akhmat & Zaman [7] studied the relationship between nuclear energy consumption, commercial energy consumption and economic growth, and found that nuclear energy consumption has a causal relationship with economic growth; commercial energy consumption and economic growth support the hypothesis of neutrality in most countries. Using a research sample of 28 developed and 34 developing countries from 1990–2017, Irfan M [8] empirically tested the two-way relationship between low-carbon energy strategies and economic growth, finding that energy efficiency promotes economic growth in both developed and developing economies, but energy diversity only promotes economic growth in developing economies; and that economic growth hinders energy efficiency in developed economies, but promotes energy efficiency in developing economies. Using data from 17 provinces in China from 2007–2010 as a research sample, Anser et al. [9] used the Data Envelopment Analysis and entropy method to study the relationship between economic growth pressure and energy efficiency and found that for every unit increase in economic growth pressure, the energy efficiency development indicator decreased by 3.4%. Using data of China from 2011–2020 as a research sample, Guan Songli and Lin Shuwei [10] found a negative relationship between energy consumption intensity and the development of the economy. Second, research on the relationship between economic growth and carbon emissions. A large number of scholars have investigated whether the relationship between the two is consistent with the hypothesis of "EKC" [11–17], and many others have investigated the interaction between the two. For example, Chen J [18] studied the two-way causality between urban CO2 emissions and economic growth, and the empirical results showed that economic growth is the Granger causality of CO2 emissions; the implementation of carbon reduction measures does not hinder economic growth. In an empirical analysis of panel data from 30 provinces in China from 2003 to 2019, Wang K, et al. [19] found a positive relationship between economic growth targets and carbon emissions, with regional heterogeneity, target heterogeneity and structural heterogeneity. Third, research on the relationship between energy, economy and carbon emissions. Apergis and Payne [20] used a panel vector error correction model to examine the relationship between economic growth, energy consumption, and CO_2_ emissions in six Central American countries and found a two-way causal relationship between energy consumption and CO_2_ emissions. Arouri et al. [21], Saboori and Sulaiman [22] examined the relationship between economic growth, energy consumption and CO2 emissions in 12 MENA countries and 5 ASEAN countries using panel unit root tests and Granger causality tests respectively, and also found a two-way causal relationship between energy consumption and carbon dioxide in the long run. Hassan Heidari et al. [23], Shen L and Sun Y [24] found a significant non-linear relationship between energy consumption, carbon dioxide emissions and economic growth. Li Y, et al. [25] conducted a study on the relationship between energy mix, digital economy and carbon emissions, which found that the impact of coal-based energy mix on carbon emissions diminishes as the digital economy develops.

Through a review of the relevant literature, we found that the studies on energy, economy and carbon emission are very common, but scholars mainly study the unidirectional influence of energy, economy and carbon emission or the correlation between the two, and some scholars also study the relationship between the three, but mostly from the perspective of the comparison between the two or the cause-effect level, and there is a lack of research on the coordination and coupling of the three, and there is a lack of research comparing the coupling and coordination of the three between different regions. In addition, most of the existing studies on the relationship between the three are based on time series data, and there are few literatures using panel data for research.

The possible contributions of this paper are: first, the existing research have mainly focused on the unidirectional influence of energy, economy and carbon emissions or the correlation between the two, but there is a lack of research on the coordinated and coupled relationship between the three. This paper provides a new research perspective by studying the coupling coordination between energy, economy and carbon emissions based on the coupling coordination degree model. Second, the existing research have mainly investigated the relationship between energy, economy and carbon emissions as a whole, but have not explored regional differences, and most of them have used regression analysis. This paper uses the Dagum Gini coefficient and decomposition method to measure the regional differences and sources of differences from the perspective of eight economic zones, which is innovative in terms of research methods and research perspectives. Third, the spatial effects of the coupling coordination development of energy-economy-carbon emissions are studied by Moran’s I index, σ convergence and β convergence, which are of great practical significance to promote the coupling coordination development and regional balanced development of energy-economy-carbon emissions in China under the background of "dual carbon".

The subsequent structure is organized as follows: the second part is research methods; the third part is results and discussions; and the last part is conclusions and recommendations.

## 2. Research design

### 2.1 Indicator system construction

A scientific and reasonable evaluation index system is a prerequisite for an accurate grasp of the level of coupling coordination development of the energy-economy-carbon emissions. Based on the principles of systematicity, scientificity, comprehensiveness and authenticity, and with reference to the research results of scholars such as Ma Huiqiang et al. [26], Yuan Lushu and Sun Xintong [27] and Wang Linyu et al. [28], this paper constructs an evaluation index system for coupling coordination development of energy-economy-carbon emissions consisting of 18 indicators (Table 1). Among them, the energy subsystem covers three aspects: total energy, energy structure and energy quality; the economic subsystem covers three aspects: economic scale, economic structure and economic quality; the carbon emission subsystem covers two aspects: carbon pollution and carbon governance.

**Table 1:** Index appraise system of coupling coordination development of energy-economy-carbon emissions.

Subsystem	Criterion	Index	Weights
Energy	Total Energy	Total energy production	47.11%
		Total energy consumption	6.28%
		Total Electricity Consumption	23.31%
	Energy Structure	Share of coal resources consumption	13.10%
	Energy Quality	Energy consumption per unit of GDP	5.63%
		Electricity consumption per unit of GDP	4.57%
Economy	Economic Scale	GDP	14.23%
		Total retail sales of consumer goods	15.54%
		Fixed asset investment	12.86%
		total export-import volume	37.09%
	Economic Structure	Share of secondary industry in GDP	2.79%
		Share of tertiary sector in GDP	6.90%
	Economic Quality	GDP Per capita	9.05%
		GDP growth rate	1.54%
Carbon Emissions	Carbon pollution	Total CO2 emissions	8.16%
		CO2 emissions per capita	7.81%
		CO2 emission intensity	11.22%
	Carbon Governance	Carbon governance investment as a percentage of GDP	72.82%

### 2.2 Research methods

**Fig 1:**
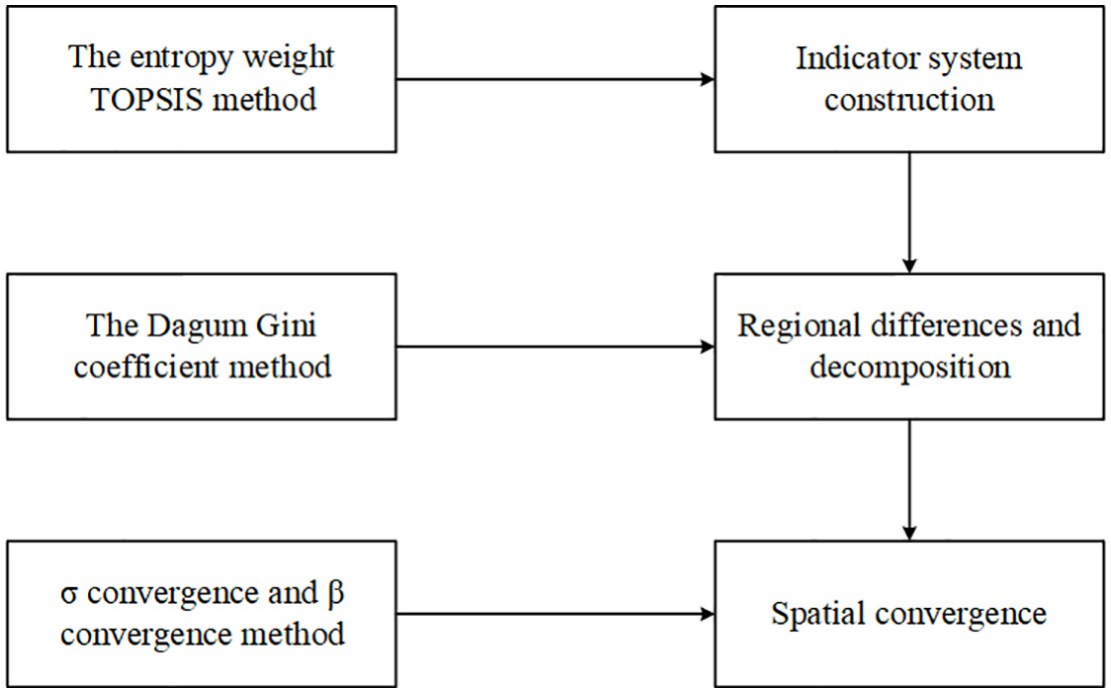
The relationship between research methods.

Fig 1 shows the relationship between research methods, that is, the entropy weight TOPSIS method is used to construct an indicator system, the Dagum Gini coefficient and decomposition method is used to measure regional differences, and σ Convergence and β Convergence method is used to measure the trend of spatial convergence.

#### 2.2.1 The entropy weight TOPSIS method

The entropy weight TOPSIS method has no special requirements for the data and has the advantages of being easy to calculate, requiring a small sample size and reasonable results, as well as being more objective for the calculation of the weights, which are added to the TOPSIS multidimensional ranking by the entropy weight calculation, making the results unaffected and more accurate. So, this paper applied the entropy TOPSIS method to measure the comprehensive development level of energy subsystem, economic subsystem and carbon emission subsystem, and then constructed the coupling degree model and coupling coordination degree model to calculate the coupling coordination degree of energy-economy-carbon emission, so as to measure the level of coupling coordination development of energy-economy-carbon emission. The steps are as follows:

In the first step, the metrics are standardized:

$$Y_{ij}=\left\{\begin{array}{c} \frac{X_{ij}-\text{min}\left(X_{ij}\right)}{\text{max}\left(X_{ij}\right)-\text{min}\left(X_{ij}\right)},X_{ij}\;is\;the\;positive\;indicator.\\ \frac{\text{max}\left(X_{ij}\right)-X_{ij}}{\text{max}\left(X_{ij}\right)-\text{min}\left(X_{ij}\right)},X_{ij}\;is\;the\;inverse\;indicator. \end{array}\right.$$ (1)

where *j* denotes the measure and *i* denotes the province; *X*_*ij*_ and *Y*_*ij*_ denote the original measure and the standardized measure, respectively; and min(*X*_*ij*_) and max(*X*_*ij*_) denote the minimum and maximum values of *X*_*ij*_, respectively.

In the second step, the information entropy *E*_*j*_ and the weights *W*_*j*_ of *Y*_*ij*_ are calculated:

$$E_{j}=ln\frac{1}{n}\sum_{i=1}^{n}\left\{\left(Y_{ij}/\sum_{i=1}^{n}Y_{ij}\right)ln\left(Y_{ij}/\sum_{i=1}^{n}Y_{ij}\right)\right\}$$ (2)

$$W_{j}=\left(1-E_{j}\right)/\sum_{j=1}^{m}\left(1-E_{j}\right)$$ (3)

In the third step, the weighting matrix *R* is constructed:

$$R={\left(r_{ij}\right)}_{n*m}$$ (4)

where *r*_*ij*_ = *W*_*j*_ * *Y*_*ij*_.

In the fourth step, the optimal solution $Q_{j}^{+}$ and the inferior solution $Q_{j}^{-}$ are developed based on the weighting matrix *R*.


$$\begin{array}{l} Q_{j}^{+}=\left(maxr_{i1},maxr_{i2},\ldots ,maxr_{im}\right)\\ Q_{j}^{-}=\left(minr_{i1},minr_{i2},\ldots ,minr_{im}\right) \end{array}$$ (5)

In the fifth step, the Euclidean distances $d_{i}^{+}$ and $d_{i}^{-}$ are calculated for each option from the optimal option $Q_{j}^{+}$ and the inferior option $Q_{j}^{-}$.


$$\begin{array}{l} d_{i}^{+}=\sqrt{\sum_{j=1}^{m}{\left(Q_{j}^{+}-r_{ij}\right)}^{2}}\\ d_{i}^{-}=\sqrt{\sum_{j=1}^{m}{\left(Q_{j}^{-}-r_{ij}\right)}^{2}} \end{array}$$ (6)

In the sixth step, the relative proximity *Z*_*i*_ of the ideal solution to the measured solution is calculated.

$$Z_{i}=\frac{d_{i}^{-}}{d_{i}^{+}+d_{i}^{-}}$$ (7)

where *Z*_*i*_ is between 0 and 1. The value of *Z*_*i*_ is a positive indicator, i.e. a larger value indicates a higher level of development of the energy-economy-carbon emission in province *i*.

In the seventh step, the coupling degree model and the coupling coordination degree model are constructed. The coupling degree model between multiple systems is expressed as:

$$C\left(Z_{1},Z_{2},\ldots ,Z_{L}\right)=n*{\left[Z_{1}Z_{2}\ldots Z_{L}/{\left(Z_{1}+Z_{2}+\cdots +Z_{L}\right)}^{L}\right]}^{1/L}$$ (8)

where L = 1, 2, 3…, M represents the number of systems. In particular, the coupling degree model between the three systems is expressed as:

$$C_{abm}=3*{\left[Z_{a}Z_{b}Z_{m}/{\left(Z_{a}+Z_{b}+Z_{m}\right)}^{3}\right]}^{1/3}$$ (9)

where *C*_*abm*_ represents the coupling value of energy-economy-carbon emissions, ranging from 0 to 1.

In order to avoid pseudo-evaluation results when energy system *Z*_*a*_, economic system *Z*_*b*_ and carbon emission system *Z*_*m*_ take small values at the same time, the coupling coordination degree model needs to be calculated on the basis of coupling degree to accurately evaluate the interaction and coordination of energy-economy-carbon emissions.

$$\begin{array}{ll} D_{abm} & =\sqrt{C_{abm}*T_{abm}}\\ T_{abm} & =\alpha Z_{a}+\beta Z_{b}+\gamma Z_{m} \end{array}$$ (10)

where *D*_*abm*_ represents the coupling coordination degree of energy-economy-carbon emission, which ranges from 0 to 1; *T*_*abm*_ is the comprehensive evaluation score of energy-economy-carbon emission; *α*, *β* and *γ* are the coefficients to be determined, which indicate the importance of the contribution of the three subsystems to the total system, and *α* + *β* + *γ* = 1. In this paper, the contribution of the three subsystems to the total system is considered equally important, so *α* = *β* = *γ* = 1/3.

#### 2.2.2 Measurement of differences

The Dagum Gini coefficient method is used to measure the intra-group and inter-group differences in the the coupling coordination degree of energy-economy-carbon emissions of the eight economic regions. Regional differences are positively related to the Gini coefficient, i.e., a larger Gini coefficient represents a larger regional difference and a lower level of coupling coordination among the provinces in the group. The specific calculation process:

$$G=\frac{\sum_{j=1}^{k}\sum_{h=1}^{k}\sum_{i=1}^{n_{j}}\sum_{r=1}^{n_{h}}\left|y_{ji}-y_{hr}\right|}{2n^{2}\bar{y}}$$ (11)

*k* is the total number of regions, which is 8 in this paper, *i* and *r* represent the number of provinces in the region, *n*_*j*_ and *n*_*h*_ represent the number of provinces in regions *j* and *h*, respectively, and *y* represents the level of coupling coordination, *n* is the total number of provinces, which is 30 in this paper, and $\bar{y}$ is the mean level of coupling coordination.

According to the decomposition theory proposed by Dagum [29], the total Gini coefficient *G* is decomposed into intra-regional differences contribution (*G*_*w*_), inter-regional differences contribution (*G*_*nb*_) and super variable density contribution (*G*_*t*_), and the relationship between the three satisfies *G* = *G*_*w*_ + *G*_*nb*_ + *G*_*t*_, which is calculated as follows:

$$\;G_{jj}=\;\frac{\frac{1}{2\bar{y}}\sum_{i=1}^{n_{j}}\sum_{r=1}^{n_{j}}\left|y_{ji}-y_{hr}\right|}{n_{j}^{2}}$$ (12)

$$\;G_{w}=\;\sum_{j=1}^{k}G_{jj}p_{j}s_{j}$$ (13)

$$\;G_{jh}=\;\frac{\sum_{i=1}^{n_{j}}\sum_{r=1}^{n_{h}}\left|y_{ji}-y_{hr}\right|}{n_{j}n_{h}\left(\bar{y_{j}}-\bar{y_{h}}\right)}$$ (14)

$$\;G_{nb}=\sum_{j=2}^{k}\sum_{h=1}^{j-1}G_{jh}\left(p_{j}s_{h}+p_{h}s_{j}\right)D_{jh}$$ (15)

$$\;G_{t}=G_{nb}=\sum_{j=2}^{k}\sum_{h=1}^{j-1}G_{jh}\left(p_{j}s_{h}+p_{h}s_{j}\right)(1-D_{jh})$$ (16)

In the above equations, *p*_*j*_ = *n*_*j*_/n, $s_{j}=n_{j}{\bar{y}}_{j}/\text{n}\;\bar{y};\;D_{jh}$ represents the relative influence of the coupling coordination degree of economic zone *j* and economic zone *h*, as shown in (17); *d*_*jh*_ is the difference of the coupling coordination degree, as shown in (18); *p*_*jh*_ is the hypervariable first order, as shown in (19) is shown; *F*_*j*_ and *F*_*h*_ are the cumulative density distribution functions in the region of economic zone *j* and economic zone *h*.


$$D_{jh}=\;\frac{d_{jh}-p_{jh}}{d_{jh}+p_{jh}}$$ (17)

$$d_{jh}=\int_{0}^{\infty }dF_{j}(y)\int_{0}^{y}(y-x)dF_{h}(x)$$ (18)

$$p_{jh}=\;\int_{0}^{\infty }dF_{h}(y)\int_{0}^{y}(y-x)dF_{j}(x)$$ (19)

#### 2.2.3 *σ* convergence and *β* convergence

The *σ* convergence is the trend of decreasing deviation of the coupling coordination degree of energy-economy-carbon emissions in different regions over time. Drawing on the research of Rezitis [30] and Xingkai Liu and Cheng Zhang [31], we used the coefficient of variation to measure the *σ* convergence of the coupling coordination degree of each region, which is calculated as follows:

$$\sigma =\frac{\sqrt{\sum_{i}^{N_{j}}(F_{ij}-\bar{F_{ij}})/N_{j}}}{\bar{F_{ij}}}$$ (20)

where *j* is the number of the eight economic zones, *i* is the number of different provinces within each economic zone, *N*_*j*_ is the number of provinces within each economic zone, and $\bar{F_{ij}}$ is the mean value of the coupling coordination degree of *j* economic zone.

*β* convergence includes two parts: absolute *β* convergence and conditional *β* convergence. Absolute *β* convergence means that the coupling coordination degree of energy-economy-carbon emissions in different regions shows a convergence trend over time without considering other factors; conditional *β* convergence means that the coupling coordination degree of energy-economy-carbon emissions in different regions converges to the respective steady-state levels over time, controlling for other factors. Meanwhile, this paper argues that there may be some spatial correlation in the coupling coordination degree of energy-economy-carbon emissions, and draws on the studies of Anselin [32] and Elhorst [33] to verify the *β* convergence process of energy-economy-carbon emission using a spatial effects model.

First, construct the absolute *β* convergence model:

$$y_{t+1}=lnEece_{i\;t+1}-lnEece_{i\;t}=\alpha +\beta_{1}lnEece_{i\;t}+\rho \omega_{ij}y_{t+1}+\delta_{1}\omega_{ij}\text{ln}Eece_{i\;t}+\mu_{i}+\lambda_{i}+\varepsilon_{it}$$ (21)

where *y*_*t*+1_ represents the change of coupling coordination in period t+1 for the ith province, *lnEece*_*i t*+1_ represents the coupling coordination level in period t+1 for the ith province, and *lnEece*_*i*_ represents the coupling coordination level in period t for the ith province. *α* is the intercept term; *β*_1_ is the convergence coefficient.

If *β*_1_ < 0, there is a convergence trend of the coupling coordination degree of energy-economy-carbon emissions; *ρ* and *δ* are the spatial effect coefficients. If both *ρ* and *δ* are 0, it is a spatial error model; *ω*_*ij*_ is the spatial weight matrix; *μ*_*i*_ and *λ*_*i*_ represent the area fixed utility and time fixed effect, respectively; *ε*_*it*_ is the error term.

Next, we constructed the conditional *β* convergence model.

$$\begin{array}{ll} y_{t+1} & =\text{ln}Eece_{i\;t+1}-\text{ln}Eece_{i\;t}=\alpha +\beta_{1}\text{ln}Eece_{i\;t}+\beta_{2}Contrls+\rho \omega_{ij}y_{t+1}+\delta_{1}\omega_{ij}\text{ln}Eece_{i\;t}\\ & +\delta_{2}\omega_{ij}Contrls+\mu_{i}+\lambda_{i}+\varepsilon_{it} \end{array}$$ (22)

where *Contrls* are control variables, and drawing on existing studies [34–37], the selected control variables include local public revenue (Rev), local public expenditure (Exp), total population (Pop), and urbanization level (Urban). Local public finance revenue is measured by the natural logarithm of local public finance revenue at year-end; local public finance expenditure is measured by the natural logarithm of local public finance expenditure at year-end; total population is measured by the natural logarithm of total population at year-end; and urbanization level is measured by the proportion of urban population to total population at year-end.

### 2.3 Data sources

With the panel data of 30 provinces in China from 2010 to 2019 as the research sample, the original data are obtained from the websites of provincial and municipal statistics bureau (https://data.stats.gov.cn/easyquery.htm?cn=E0103), China Statistical Yearbook (http://www.stats.gov.cn/tjsj/ndsj/), CSMAR database (https://www.gtarsc.com/) and Wind database (https://www.wind.com.cn/NewSite/default.html).

## 3. Research results

### 3.1 Characterization of coupling coordination development of energy-economy-carbon emissions

#### 3.1.1 Comprehensive evaluation of the energy-economy-carbon emissions subsystem

**Fig 2:**
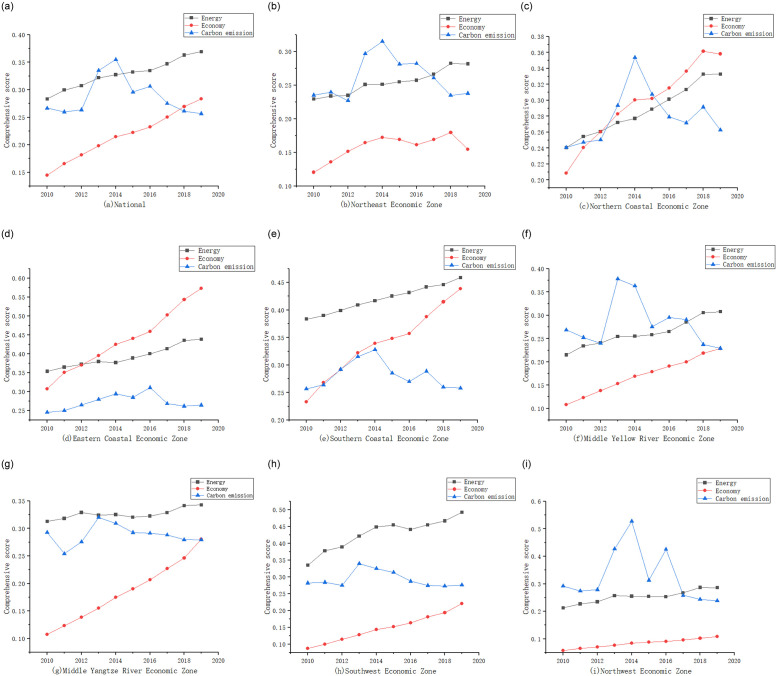
Comprehensive level of energy-economy-carbon emission subsystems in China and the eight economic zones.

Based on the entropy TOPSIS method, we calculated the comprehensive level of the energy-economy-carbon emissions subsystem of the whole country and the eight economic regions in turn, and Fig 2 shows the development trends of the three subsystems. As can be seen in Fig 2(a), the energy system has the highest composite score and the economic system has the lowest composite score; overall, the composite scores of the energy and economic systems show a steady upward trend from 2010 to 2019, indicating a good development trend of the energy and economic systems in China in the last decade, which is consistent with previous studies. The composite score of the carbon emissions system shows a large fluctuation, with an increasing trend before 2014 and a decreasing trend from 2015. This is mainly because on the one hand, 2015 is the closing year of China’s 12th Five-Year Plan, driven by mass entrepreneurship and innovation, the economy has maintained high speed growth and gradually advanced to the middle and high-end level, resulting in an overall increase in GDP; on the other hand, China has comprehensively implemented the new development concept of innovation, coordination, green, openness and sharing since 2015, the level of carbon pollution has gradually decreased and the amount of investment in carbon control has gradually increased, the increase in the amount of investment in carbon control is much lower than the increase in GDP, resulting in a downward trend in the proportion of investment in carbon control to GDP. According to Table 1, the proportion of carbon governance is 72.82% and the proportion of carbon pollution is 27.18%, so the integrated level of carbon emissions not only did not improve after 2015, but also showed a downward trend. This shows that the situation of carbon management in China is still serious, which illustrates the necessity and urgency for China to vigorously promote " peak carbon dioxide emissions " and " carbon neutrality ".

Fig 2(b)–2(i) show the integrated level of energy-economy-carbon emissions system in the eight economic regions, which on the whole is in line with national trends. Specifically, the economic level of the Eastern Coastal Economic Zone is the highest and maintains continuous growth. The economic level of the the Northern Coastal Economic Zone, Southern Coastal Economic Zone, Middle Yellow River Economic Zone, Middle Yangtze River Economic Zone and Southwest Economic Zone maintains fast growth, while the economic level of the Northeast Economic Zone and Northwest Economic Zone is slower. The development trends of carbon emissions in the Eastern Coastal Economic Zone, the Middle Yangtze River Economic Zone, and the Southwest Economic Zone are relatively stable, thus showing that the fluctuations in the carbon emissions system are mainly brought about by the other five economic zones. The energy system in the Northern Coastal Economic Zone and the Southern Coastal Economic Zone maintains a faster growth trend.

#### 3.1.2 The overall characteristics of the coupling coordination development of energy-economy-carbon emissions

Drawing on relevant studies [38–40] and based on the magnitude of the coupling coordination degree of energy-economy-carbon emission, a uniform distribution function method is used to classify the intervals and levels of coupling coordination. The specific classification is shown in Table 2.

**Table 2:** Classification of coupling coordination levels.

Coupling coordination degree (D)	Coupling Coordination Levels	Coupling coordination degree (D)	Coupling Coordination Levels
0<D<0.1	Extreme disorder	0.5≤D<0.6	Barely coordination
0.1≤D<0.2	Severe disorder	0.6≤D<0.7	Primary Coordination
0.2≤D<0.3	Moderate disorder	0.7≤D<0.8	Intermediate coordinate
0.3≤D<0.4	Mild disorder	0.8≤D<0.9	Good coordination
0.4≤D<0.5	Imminent disorder	0.9≤D<1	Quality Coordination

**Fig 3:**
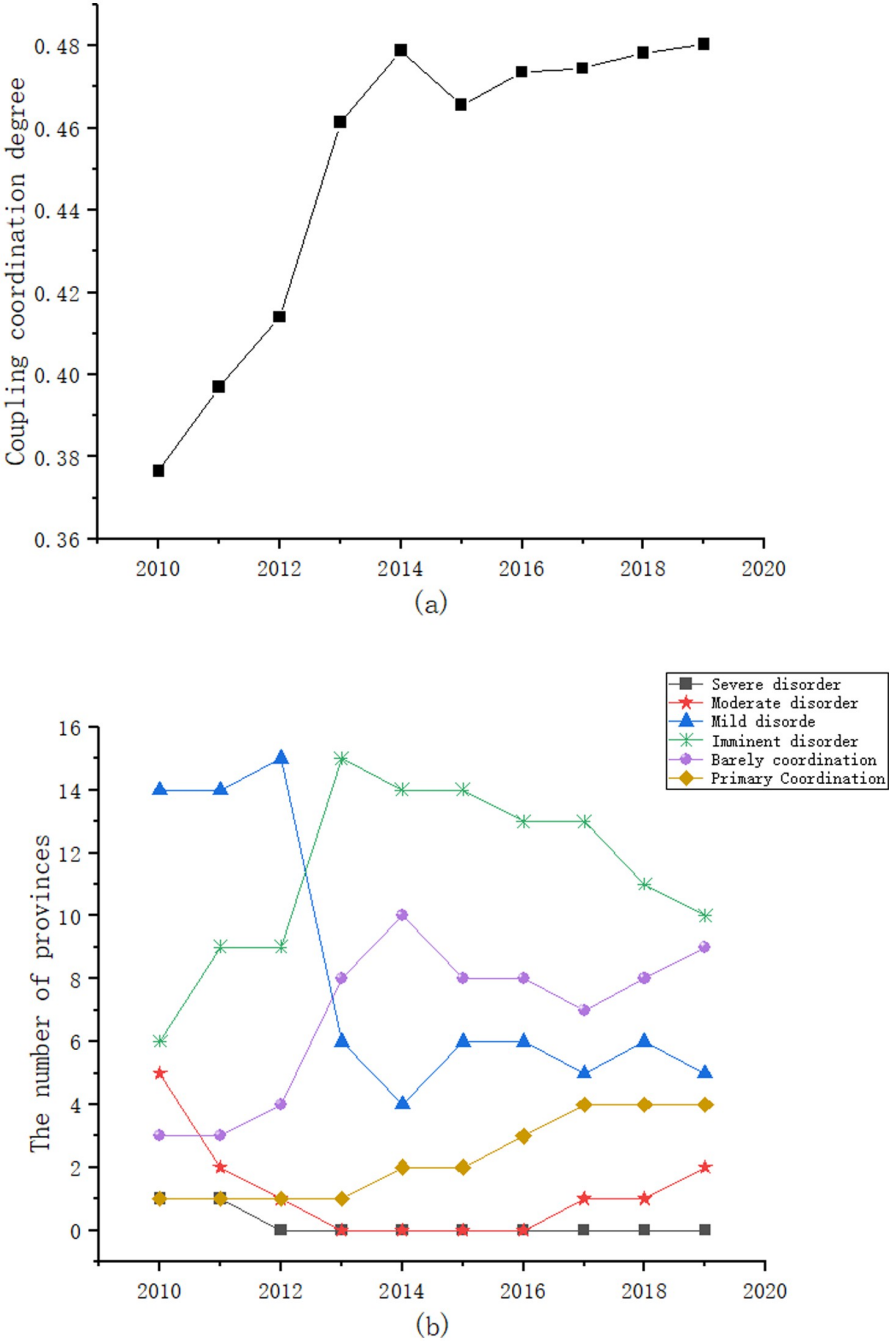
Trends in the coupling coordination development of the energy-economy-carbon emission system in China.

Based on the coupling coordination degree measurement model, the coupling coordination degree of energy-economy-carbon emission system is calculated by using the comprehensive scores of energy system, economic system and carbon emission system of 30 provinces in China. Table 3 presents the hierarchy of the coupled coordination relationship of the national energy-economy-carbon emission from 2010 to 2019. Fig 3(a) illustrates the trend of the coupled coordination relationship of the energy-economy-carbon emission and Fig 3(b) shows the trend of the number of provinces in various coupling coordination relationships each year. From Fig 3(a), it can be seen that, firstly, the coupling coordination of the energy-economy-carbon emission from 2010 to 2019 has been increasing and has obvious phase characteristics, with a faster growth rate before 2013 and a gradual slowdown after 2013; secondly, the coupling coordination of the energy-economy-carbon emission system in China is still at the stage of imminent disorder. Therefore, it is necessary to vigorously promote the coupling coordination degree of energy-economy-carbon emission system, especially to continuously increase the investment in carbon governance, so as to better realize the coordinated development of energy-economy-carbon emission system. From Fig 3(b), it can be seen that, firstly, the coupling coordination of the energy-economy-carbon emission system in 30 provinces mainly includes six levels: severe disorder, moderate disorder, mild disorder, imminent disorder, barely coordination and primary coordination; secondly, the highest number of provinces were at the two levels of mild disorder and imminent disorder before 2013, and the highest number of provinces were at the two levels of imminent disorder and barely coordination after 2013. This may be due to the change in economic growth targets proposed at the 2013 Central Economic Work Conference, where the word “fast” disappeared and “sustainable and healthy development” became the new target for economic growth. The provinces have increased their investment in energy conservation and emission reduction while focusing on economic development, and as a result, the level of coupling coordination of the energy-economy-carbon emission system has gradually increased since 2013.

**Table 3:** Coupling coordination degree of the national energy-economy-carbon emission system from 2010 to 2019.

Year	Coupling coordination degree	Coupling Coordination Levels	Year	Coupling coordination degree	Coupling Coordination Levels
2010	0.377	mild disorder	2015	0.466	imminent disorder
2011	0.397	mild disorder	2016	0.474	imminent disorder
2012	0.414	imminent disorder	2017	0.475	imminent disorder
2013	0.461	imminent disorder	2018	0.478	imminent disorder
2014	0.479	imminent disorder	2019	0.480	imminent disorder

#### 3.1.3 Regional characterization of the coupling coordination development of energy-economy-carbon emission system

**Fig 4:**
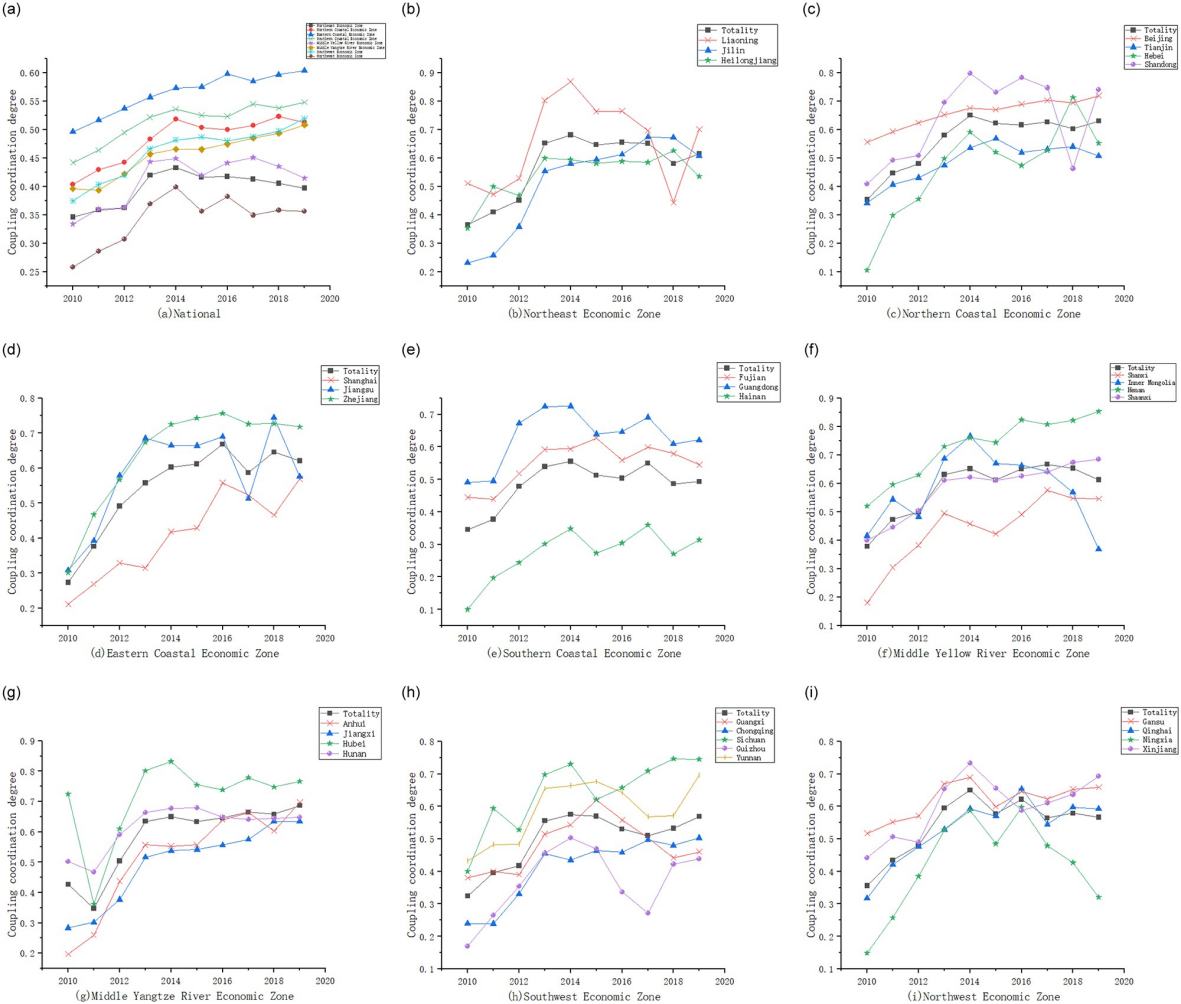
Coupling coordination degree of energy-economy-carbon emission systems within the eight economic regions.

Fig 4 shows the coupling coordination degree of the energy-economy-carbon emission systems of the eight economic regions and the internal provinces, respectively. As can be seen in Fig 4(a), the coupling coordination of the energy-economy-carbon emissions system in the eight economic regions of China from 2010 to 2019 is increasing and has obvious regional heterogeneity. The coupling coordination degree of the Eastern Coastal Economic Zone is the highest, with a mean value of 0.564, and its coupling coordination degree reaches 0.604 in 2019, i.e., it gradually improves from the stage of imminent disorder to the stage of primary coordination. The Northwest Economic Zone has the lowest coupling coordination degree with a mean value of 0.342, and its coupling coordination degree is only 0.357 in 2019, which is still in the stage of mild disorder. In addition, the coupling coordination degree of the Northern Coastal Economic Zone, Eastern Coastal Economic Zone, Southern Coastal Economic Zone, Middle Yangtze River Economic Zone and Southwest Economic Zone is higher than the national average, while the coupling coordination degree of the Northeast Economic Zone, the Middle Yellow River Economic Zone and the Northwest Economic Zone is lower than the national average, especially the Northeast Economic Zone and Northwest Economic Zone, which are even at the stage of mild disorder. Fig 4(b) presents the coupling coordination degree of the energy-economy-carbon emissions within the Northeast Economic Zone and the internal provinces. The coupling coordination degree within the Northeast Economic Zone basically shows a fluctuating upward trend, among which the coupling coordination degree of Jilin and Heilongjiang provinces has a small change and shows a steady growth, and the coupling coordination degree of Liaoning province shows a large fluctuation. This indicates that the low coupling coordination degree of Northeast economic zone is mainly caused by Liaoning province. Equipment manufacturing, metallurgy, petrochemicals and agricultural products are the four pillar industries of Liaoning province, which are high energy-consuming industries, especially metallurgy and petrochemicals, with high energy consumption and high levels of carbon emissions. With the promotion of sustainable development and high-quality economic development, Liaoning Province is also promoting economic transformation, with significant changes in energy consumption and carbon emission, and therefore large fluctuations in the level of the coupling coordination degree of the energy-economy-carbon emissions system. Fig 4(c) shows the coupling coordination degree of the energy-economy-carbon emissions system within the Northern Coastal Economic Zone and the internal provinces. The coupling coordination degree within the Northern Coastal Economic Zone basically shows a fluctuating upward trend, among which the coupling coordination degree of Beijing and Tianjin has less change and shows a steady growth, the coupling coordination degree of Hebei Province and Shandong Province shows large fluctuations and the trend of change in the two is completely opposite after 2015. Fig 4(d) shows the coupling coordination degree of the energy-economy-carbon emissions system within the Eastern Coastal Economic Zone and the internal provinces. The coupling coordination degree within the Eastern Coastal Economic Zone basically shows a fluctuating upward trend, with less fluctuation before 2016. After 2016, it fluctuates more, especially in Jiangsu Province, which shows stronger fluctuation after 2016. This may be related to a series of coal power ultra-low emission and energy efficiency retrofit policies issued by Jiangsu Province in 2016. Fig 4(e) shows the coupling coordination degree of the energy-economy-carbon emission system within the Southern Coastal Economic Zone and the internal provinces. The coupling coordination degree within the Southern Coastal Economic Zone and the internal provinces basically maintains a consistent trend. Fig 4(f) shows the coupling coordination degree of the energy-economy-carbon emission system within the Middle Yellow River Economic Zone and the internal provinces. The coupling coordination degree within the Middle Yellow River Economic Zone and provinces of Shanxi, Shaanxi and Henan basically shows a fluctuating upward trend and less fluctuation; the coupling coordination degree of Inner Mongolia Autonomous Region shows a large fluctuation, with a sharp increase before 2014 and a sharp decrease after 2014. Fig 4(g) shows the coupling coordination degree of energy-economy-carbon emission system within the Middle Yangtze River Economic Zone and the internal provinces. The coupling coordination degree within the Middle Yangtze River Economic Zone has been increasing since 2011, and the coupling coordination degree of each province within the zone has gradually converged, which is closely related to the national efforts to promote the integration of "Yangtze River Delta". Fig 4(h) shows the coupling coordination degree of the energy-economy-carbon emission system within the Southwest Economic Zone and the internal provinces. The coupling coordination degree within the Southwest Economic Zone basically shows a fluctuating upward trend, among which the coupling coordination degree of Chongqing has been maintaining a stable growth trend; the coupling coordination degree of Guizhou Province, Sichuan Province, Yunnan Province and Guangxi Zhuang Autonomous Region fluctuates more. Fig 4(i) shows the coupling coordination degree of the energy-economy-carbon emission system within the Northwest Economic Zone and the internal provinces. The coupling coordination degree within the Northwest Economic Zone is also generally fluctuating and increasing, while we find that the coupling coordination degree of Ningxia Hui Autonomous Region shows large fluctuations, and the coupling coordination degree of Gansu Province, Qinghai Province and Xinjiang Uyghur Autonomous Region is more stable and gradually converging.

### 3.2 Regional differences and decomposition of the coupling coordination development of energy-economy-carbon emission system

The Dagum Gini coefficient and decomposition method is used to calculate the overall differences in the coupling coordination degree of the energy-economy-carbon emission system of the eight economic regions from 2010 to 2019, while the overall differences are decomposed.

#### 3.2.1 Overall difference

**Fig 5:**
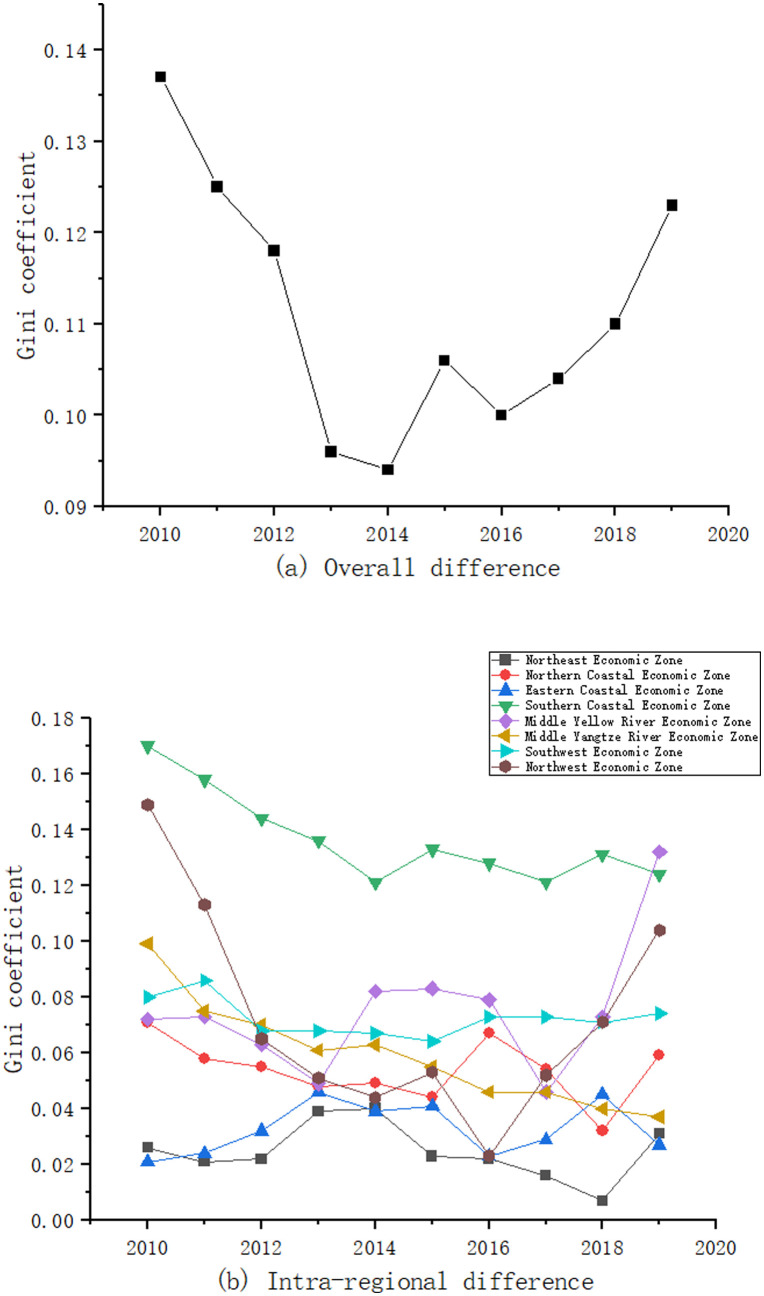
Overall and intra-regional differences of coupling coordination degree of energy-economy-carbon emission system.

Fig 5(a) shows the trend of the overall Gini coefficient of coupling coordination degree of energy-economy-carbon emission system from 2010 to 2019. From 2010 to 2014, the Gini coefficient decreased from 0.137 to 0.094 with a decrease of 31.39%. From 2014 to 2019, the Gini coefficient increased from 0.094 to 0.123 with an increase of 30.85%. It can be seen that the overall difference of the coupling coordination degree of energy-economy-carbon emission system in China shows a decreasing and then increasing trend, which is consistent with the previous study. The overall difference of the coupling coordination degree of energy-economy-carbon emission system is mainly caused by the development trend of the carbon emissions. The reason for the shrinking overall difference until 2014 may be that after the Great Recession in 2008, countries began to develop their economies vigorously at all costs, and China was no exception. Each province pushed hard for economic development, while all neglected to protect the environment, leading to an increase in energy consumption and a sharp rise in carbon emissions. As a result, the level of the coupling coordination degree of energy-economy-carbon emission system is low and growing slowly in all provinces and cities, so the differences are shrinking. Therefore, in order to realize the coupling coordination development of energy-economy-carbon emission system, it is necessary to focus on increasing the proportion of investment in carbon control while reducing carbon pollution.

#### 3.2.2 Intra-regional difference

Fig 5(b) gives the trend of the Gini coefficient within the eight economic regions. The differences of the coupling coordination degree of the energy-economy-carbon emission system in the eight economic regions show significant regional and phased characteristics. Specifically, the intra-regional difference of coupling coordination degree in the Northeast Economic Zone is the smallest during the sample examination period, with a general trend of decreasing, then increasing and then gradually decreasing, especially since 2014 when the intra-regional difference has been decreasing, with a decrease of 82.5%. The intra-regional difference of coupling coordination degree in the Southern Coastal Economic Zone is the largest, but shows a gradual downward trend from 0.170 in 2010 to 0.124 in 2019, with a decrease of 27.06%. The intra-regional difference of coupling coordination degree in the Middle Yangtze River Economic Zone shows a gradual decline, from 0.099 in 2010 to 0.037 in 2019. The Northwest Economic Zone is the region with the largest intra-regional difference in coupling coordination degree among the eight economic zones, showing an overall trend of a sharp decline followed by a sharp rise. There is also considerable intra-regional difference of the coupling coordination degree in the Middle Yellow River Economic Zone. Therefore, it is necessary to increase the coupling coordination degree of the Northwest Economic Zone and the Middle Yellow River Economic Zone.

#### 3.2.3 Inter-regional difference

**Fig 6:**
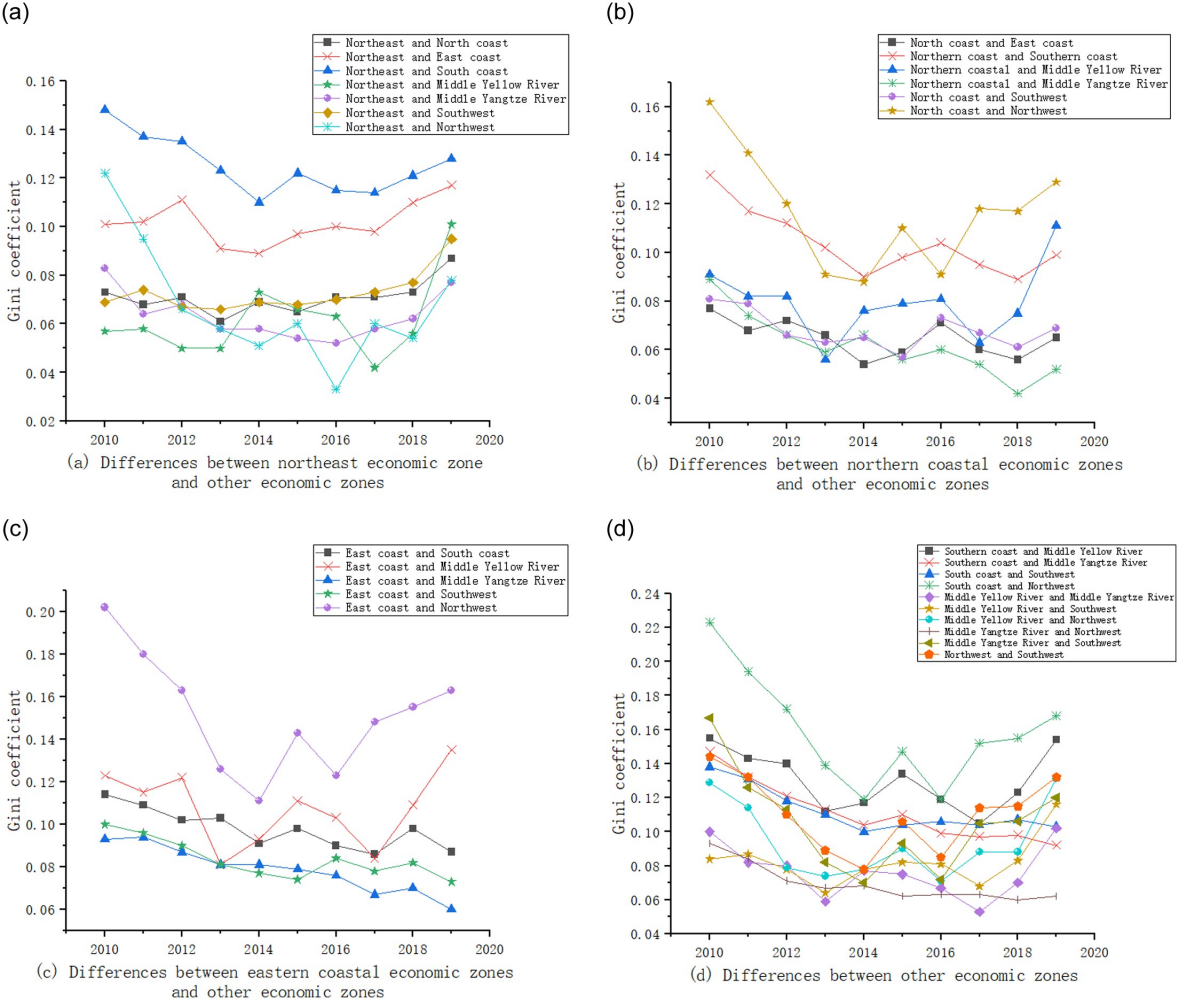
Inter-regional difference in the coupling coordination degree of the energy-economy-carbon emission system.

Fig 6 shows the trend of inter-regional differences in the eight economic zones after the pairing. As can be seen from Fig 6(a), firstly, the inter-regional difference in the coupling coordination degree between the Northeast Economic Zone and the Southern Coastal Economic Zone is the largest, and the inter-regional difference in the coupling coordination degree with the Middle Yellow River Economic Zone is the smallest. Secondly, the inter-regional difference in the coupling coordination degree between the Northeast Economic Zone and the Southern Coastal Economic Zone, the Northwest Economic Zone and the Middle Yangtze River Economic Zone shows a decreasing and then increasing trend. Thirdly, the inter-regional difference in the coupling coordination degree between the Northeast Economic Zone and the Eastern Coastal Economic Zone, the Northern Coastal Economic Zone, the Southwest Economic Zone and the Middle Yellow River Economic Zone shows a fluctuating increasing trend. As can be seen from Fig 6(b), firstly, the inter-regional difference in the coupling coordination degree between the Northern Coastal Economic Zone and the Northwest Economic Zone is the largest, and the inter-regional difference in the coupling coordination degree with the Middle Yangtze River economic zone is the smallest. Secondly, the inter-regional difference in the coupling coordination degree between the Northern Coastal Economic Zone and the Southern Coastal Economic Zone, the Eastern Coastal Economic Zone, the Middle Yangtze River Economic Zone and the Southwest Economic Zone shows a fluctuating decreasing trend. Thirdly, the inter-regional difference in the coupling coordination degree between the Northern Coastal Economic Zone and the Northwest Economic Zone and the Middle Yellow River Economic Zone shows a trend of first decreasing and then fluctuating upwards. As can be seen from Fig 6(c), firstly, the inter-regional difference in coupling coordination degree between the Eastern Coastal Economic Zone and the Northwest Economic Zone is the largest, and the inter-regional difference in coupling coordination degree with the Middle Yangtze River Economic Zone is the smallest. Secondly, the inter-regional difference of coupling coordination degree between the Eastern Coastal Economic Zone and the Southern Coastal Economic Zone, the Middle Yangtze River Economic Zone and the Southwest Economic Zone shows a decreasing trend. Thirdly, the inter-regional difference in coupling coordination degree between the Eastern Coastal Economic Zone and the Northwest Economic Zone and the Middle Yellow River Economic Zone shows a trend of first decreasing and then fluctuating upwards. As can be seen from Fig 6(d), among the remaining other economic zones, the inter-regional difference in the coupling coordination degree between the South Coastal Economic Zone and the Northwest Economic Zone is the largest, and the inter-regional difference in the coupling coordination degree between the Middle Yangtze River Economic Zone and the Northwest Economic Zone is the smallest.

In summary, the inter-regional difference in the coupling coordination degree of the energy-economy-carbon emission system in China is mainly caused by the inter-regional difference. Therefore, to realize the coupling coordination development of energy-economy-carbon emission system, it is necessary to focus on reducing the inter-regional difference among economic zones, especially the inter-regional difference between the Northwest Economic Zone, the Middle Yellow River Economic Zone and the Coastal Economic Zone.

#### 3.2.4 Source of differences

**Fig 7:**
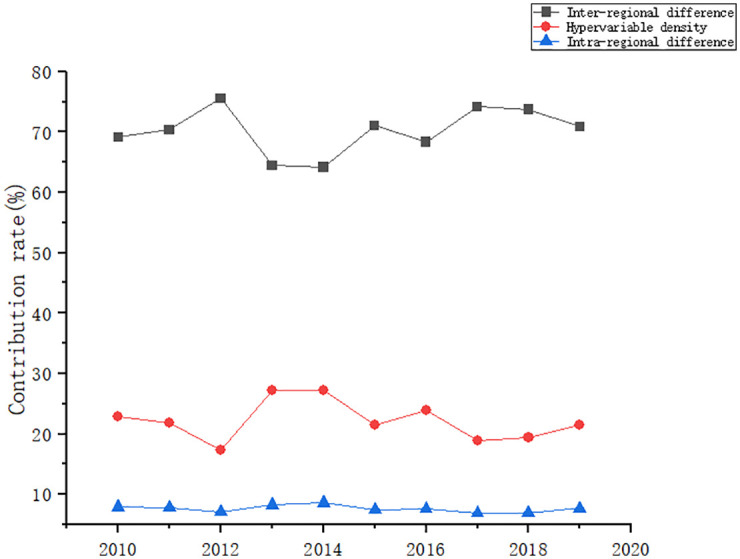
Differential contribution of coupling coordination degree of energy-economy-carbon emission system.

Fig 7 gives the inter-regional variance contribution, intra-regional variance contribution and hypervariable density contribution of coupling coordination degree of the energy-economy-carbon emission system from 2010 to 2019. The contribution of inter-regional difference in the coupling coordination degree of the energy-economy-carbon emission system in China is the largest and has been dominant since 2010, followed by the contribution of hypervariable density, and the contribution of intra-regional differences is the smallest, indicating that the main source of differences in the coupling coordination degree of the energy-economy-carbon emission system is inter-regional difference. Therefore, to realize the coupling coordination development of energy-economy-carbon emission system, it is necessary to focus on reducing inter-regional difference while taking into account the reduction of intra-regional difference.

### 3.3. Spatial convergence of the coupling coordination development of energy-economy-carbon emission system

#### 3.3.1 Spatial correlation test

Through the analysis of the coupling coordination degree among provinces within the eight economic regions, it is initially found that there is a certain regional spatial effect on the coupling coordination degree of the energy-economy-carbon emission system in China. To further test the spatial interaction effect of energy-economy-carbon emission systems in different regions, Moran’s I index is used referring to the related research [41–45]. The results are shown in Table 4. The spatial interaction effect of the coupling coordination degree of energy-economy-carbon emission system is not significant before 2015, and only the Moran’s I index in 2012 is significantly positive at the 5% level. The Moran’s I index is significantly positive after 2015, and the significance of the index is increasing, and the index is gradually becoming larger, indicating that the coupling coordination degree of energy-economy-carbon emission system shows a trend of "high-high aggregation and low-low aggregation" after 2015, i.e., there is a spatially positive correlation, which gradually increases over time.. Therefore, in order to realize the coupling coordination development of energy-economy-carbon emission system, it is necessary to strengthen the driving effect of regions with higher coupling coordination degree on neighboring regions.

**Table 4:** Spatial correlation of the coupling coordination development of energy-economy-carbon emission system.

Year	Moran’s I	Year	Moran’s I
2010	0.092	2015	0.112*
	(0.124)		(0.093)
2011	0.037	2016	0.133*
	(0.259)		(0.067)
2012	0.169**	2017	0.170**
	(0.032)		(0.033)
2013	-0.038	2018	0.203**
	(0.485)		(0.016)
2014	-0.080	2019	0.356***
	(0.340)		(0.000)

Note:

*, **, *** represent the significance levels of 10%, 5% and 1%, respectively; the values in brackets are the corresponding z statistics.

#### 3.3.2 σ convergence analysis

**Fig 8:**
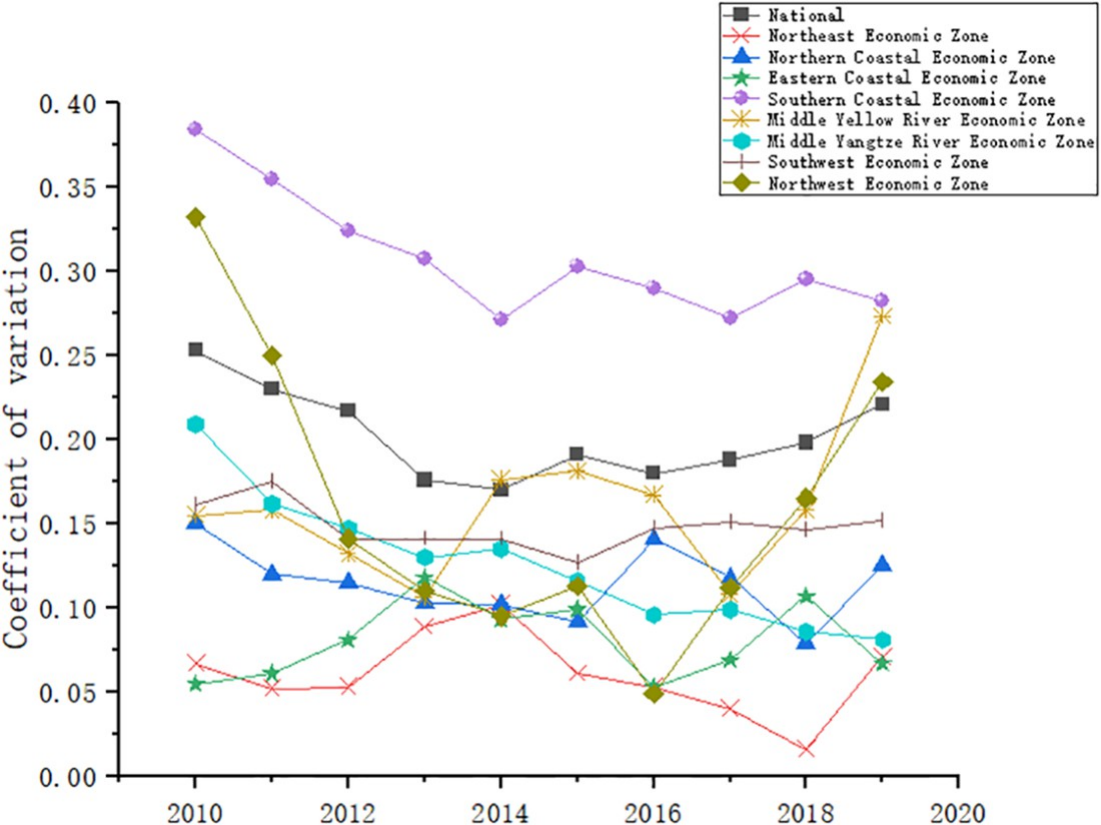
σ convergence of the coupling coordination degree of the energy-economy-carbon emission system.

Fig 8 shows the trend of the coefficient of variation of the coupling coordination degree of the energy-economy-carbon emission system for the whole country and the eight economic regions from 2010 to 2019. At the national level, the coefficient of variation shows a gradually decreasing trend before 2014 and a gradually increasing trend after 2014, i.e., the difference in the coupling coordination degree of the energy-economy-carbon emission at the national level become progressively larger after 2014 without σ convergence, which is consistent with previous discussions. At the regional level, the coefficient of variation of the Southern Coastal Economic Zone and the Middle Yangtze River Economic Zone shows a gradually decreasing trend, indicating that there is a σ convergence in the coupling coordination degree of the energy-economy-carbon emission systems. This may be related to the implementation of strategies such as the integration of the Yangtze River Delta, the construction of the Yangtze River Economic Belt and Guangdong-Hong Kong-Macao greater bay area. The coefficient of variation of the Northwest Economic Zone and the Middle Yellow River Economic Zone has an obvious trend of decreasing and then increasing, and has increased sharply in the past three years, indicating that there is noσ convergence in the coupling coordination degree of the energy-economy-carbon emission system of the Northwest Economic Zone and the Middle Yellow River Economic Zone This may be due to the fact that the two economic zones, as China’s largest bases for coal mining and deep processing, natural gas and hydro energy development, iron and steel industry, dairy industry, non-ferrous metal industry and strategic energy replacement bases, have a complex industrial composition, so that with the introduction of the “dual carbon”, the response and implementation speed of the provinces in the region varies considerably, and therefore the coupling coordination degree varies continuously. There is a slight trend of decreasing and then increasing coefficient of variation for the Southwest Economic Zone, indicating that there is no σ-convergence in the coupling coordination degree in the Southwest Economic Zone. The coefficient of variation of the Northern Coastal Economic Zone shows a significant decreasing trend until 2015, and fluctuates more after 2015 without significant σ convergence; the coefficient of variation of the Eastern Coastal Economic Zone and the Northeastern Economic Zone also fluctuates more, without significant σ convergence.

#### 3.3.3 β convergence analysis

Absolute β convergence. Table 5 shows the results of the model applicability test. The absolute β convergence rejects the original hypothesis that the SDM model can be reduced to the SAR model, but does not reject the original hypothesis that the SDM model can be reduced to the SEM model, and the Hausman test again rejects the original hypothesis of random effects, so the SEM model with fixed effects is used to test the absolute β convergence.

**Table 5:** Model applicability test.

Test		Absolute β convergence		Conditional β convergence	
		Statistics	P-value	Statistics	P-value
LR test	LR test (SAR)	3.65	0.056	39.71	0.000
	LR test (SEM)	0.27	0.601	33.78	0.000
Wald test	Wald test (SAR)	3.84	0.050	43.38	0.000
	Wald test (SEM)	0.31	0.576	35.75	0.000
Hausman test		FE		FE	

Table 6 shows that the β convergence coefficients of the coupling coordination degree of energy-economy-carbon emission system of both the national and eight economic regions are significantly positive at the 1% level, indicating that there is no β convergence and that it is a significant divergence process, i.e., the regional difference of the coupling coordination degree of energy-economy-carbon emission system keeps expanding as time advances. Meanwhile, by comparing the magnitude of the coefficient of the coupling coordination degree of the energy-economy-carbon emission system, it is found that the difference expands fastest in the Southern Coastal Economic Zone, followed by the Middle Yangtze River economic zone, and the difference expands slowest in the Northeast Economic Zone.

**Table 6:** Absolute β convergence of the coupling coordination degree of the energy-economy-carbon emission system.

Variables	National	Northeast Economic Zone	Northern Coastal Economic Zone	Eastern Coastal Economic Zone	Southern Coastal Economic Zone	Middle Yellow River Economic Zone	Middle Yangtze River Economic Zone	Southwest Economic Zone	Northwest Economic Zone
Eece	0.766***	0.550***	0.774***	0.996***	1.108***	0.618***	1.048***	0.888***	0.881***
	(12.71)	(2.80)	(3.31)	(7.97)	(7.95)	(4.65)	(14.20)	(4.96)	(5.27)
lambda	0.396***	0.414***	0.284	0.032	0.156	0.348***	0.423***	0.593***	0.269**
	(6.08)	(3.21)	(1.25)	(0.15)	(1.27)	(2.65)	(3.68)	(4.10)	(2.42)
R^2^	0.568	0.430	0.598	0.761	0.761	0.352	0.887	0.856	0.495
LogL	371.119	45.646	54.596	60.521	53.909	34.619	85.513	98.469	28.977

Conditional β convergence. Considering that there are large differences in other social characteristics between different regions, there will be subject to some errors using only absolute β convergence, so conditional β convergence analysis is conducted. The results of the model applicability test in Table 5 show that the conditional β convergence rejects the original hypothesis that the SDM model can be reduced to a SAR model or a SEM model respectively, and the Hausman in turn test rejects the original hypothesis of random effects, so the conditional β convergence of the coupling coordination of the energy-economy-carbon emission system is tested using the fixed effects SDM model.

Table 7 shows the results of the tests for the conditional β convergence of the coupling coordination degree of the energy-economy-carbon emission system in the national and eight economic regions under the spatial Durbin model. The β convergence changes significantly after the inclusion of other influencing factors. The coefficients of the coupling coordination degree of energy-economy-carbon emission system in the national, Southern Coastal Economic Zone, Middle Yangtze River Economic Zone and Southwest Economic Zone are significantly positive at the 10% level, indicating that there is no conditional β convergence in the coupling coordination degree of energy-economy-carbon emission system. And it is a significant diverging trend, which indicates that the better coupled areas continue to develop at a faster pace and that the differences with the less coupled areas are gradually increasing. The coefficients of the Northeast Economic Zone and North Coastal Economic Zone are negative but insignificant at the 10% level, indicating that there is no conditional β convergence in the Northeast Economic Zone and North Coastal Economic Zone. The coefficients of the Eastern Coastal Economic Zone, the Middle Yellow River Economic Zone, and the Northwest Economic Zone are positive and insignificant at the 10% level, indicating that the convergence or divergence trend of the coupling coordination degree of energy-economy-carbon emission system is not significant. At the same time, by comparing the absolute values of the convergence coefficients, it is found that the coupling coordination degree of energy-economy-carbon emission system in the Middle Yangtze River Economic Zone changes the fastest, i.e., the coupling coordination degree of energy-economy-carbon emission system in the Middle Yangtze River Economic Zone develops rapidly towards a better level. Therefore, while promoting the coupling coordination development of the energy-economy-carbon emission system in the Middle Yangtze River Economic Zone, it is more important to focus on the coupling coordination development of Northeast Economic Zone, Northern Coastal Economic Zone, Eastern Coastal Economic Zone, Middle Yellow River Economic Zone and Northwest Economic Zone.

**Table 7:** Conditional β convergence of the coupling coordination of the energy-economy-carbon emission system.

Variables	National	Northeast Economic Zone	Northern Coastal Economic Zone	Eastern Coastal Economic Zone	Southern Coastal Economic Zone	Middle Yellow River Economic Zone	Middle Yangtze River Economic Zone	Southwest Economic Zone	Northwest Economic Zone
Eece	0.444***	-0.044	-0.120	0.163	0.319*	0.083	0.857***	0.355**	0.125
	(7.54)	(-0.20)	(-0.72)	(0.91)	(1.84)	(0.50)	(6.62)	(2.41)	(0.71)
Rev	0.161***	-0.072	0.571***	0.304***	0.288*	0.109	-0.227**	0.203**	0.086
	(2.66)	(-0.42)	(2.76)	(2.70)	(1.69)	(0.44)	(-2.07)	(1.97)	(0.17)
Exp	-0.079	0.126	-0.425*	-0.151	-0.223	1.071**	0.018	-0.133	0.503
	(-0.97)	(0.43)	(-1.85)	(-1.28)	(-1.58)	(2.29)	(0.10)	(-1.14)	(1.30)
Pop	0.126	4.669*	-1.325***	-1.592	2.825**	16.085***	3.158**	0.617	1.284
	(0.49)	(1.88)	(-3.62)	(-1.37)	(2.17)	(3.47)	(2.16)	(0.62)	(1.13)
Urban	0.124	3.877*	0.053	1.288	3.403***	-0.251	0.811	-1.111	-1.510
	(1.05)	(1.76)	(0.45)	(1.28)	(3.56)	(-0.38)	(0.79)	(-1.20)	(-1.07)
W*Eece	-0.159*	0.011	0.015	0.399	0.189	0.223	0.015	-0.122	-0.370**
	(-1.80)	(0.04)	(0.07)	(1.34)	(1.26)	(0.92)	(0.04)	(-0.65)	(-2.34)
W*Rev	0.279***	0.136	0.359	0.593***	0.536***	0.739	0.528***	0.182	1.526**
	(3.22)	(0.68)	(1.39)	(3.80)	(3.32)	(1.28)	(3.84)	(1.26)	(2.51)
W*Exp	-0.256**	-0.357	0.059	-0.594***	-0.447***	-2.520*	-0.533**	-0.121	-0.821*
	(-2.43)	(-0.90)	(0.23)	(-3.98)	(-3.33)	(-1.84)	(-2.10)	(-0.78)	(-1.85)
W* Pop	2.162***	4.126	-1.184*	-0.678	-4.006**	-3.835	6.760	7.774*	3.970
	(4.25)	(1.16)	(-1.78)	(-0.30)	(-2.39)	(-0.27)	(1.42)	(1.67)	(1.16)
W*Urban	-0.173	2.924	0.053	2.417	-1.607	1.992	-3.324	-2.158	-9.084***
	(-0.64)	(1.14)	(0.45)	(1.21)	(-1.62)	(1.33)	(-0.93)	(-1.21)	(-2.80)
ρ	0.118*	0.250*	0.105	-0.382*	-0.080	0.383***	0.403***	0.374***	-0.173
	(1.71)	(1.85)	(0.69)	(-1.87)	(-0.63)	(3.05)	(3.36)	(2.93)	(-1.31)
R^2^	0.707	0.750	0.832	0.929	0.925	0.646	0.928	0.928	0.778
LogL	414.744	56.075	71.664	80.410	70.381	47.773	94.077	111.858	43.201

## 4. Conclusions and recommendations

Based on the panel data of 30 provinces in China from 2010 to 2019, this paper measured the coupling coordination degree of energy-economy-carbon emission systems in eight economic regions and analyzed its regional differences and spatial convergence. The conclusions are as follows: First, the level of energy system and economic system in the three systems show a steady upward trend, while the level of carbon emission system shows a large fluctuation and a downward trend in the last five years; by region, the Northern Coastal Economic Zone and the Southern Coastal Economic Zone maintain a fast growth trend in energy, the Eastern Coastal Economic Zone has the highest economic level and maintains a continuous growth trend, the trends of carbon emissions in the Eastern Coastal Economic Zone, the Middle Yangtze River Economic Zone and the Southwest Economic Zone are relatively stable. Second, the coupling coordination degree of energy-economy-carbon emission system is improving and and is characterised by distinct phases, but it is still on the verge of imminent disorder; by region, the coupling coordination degree of energy-economy-carbon emission system in the Eastern Coastal Economic Zone is the highest, while the coupling coordination degree in the Northwest Economic Zone is the lowest. Third, the overall difference in the coupling coordination degree of energy-economy-carbon emission system in China shows a decreasing trend followed by an increasing trend and regional characteristics; the inter-regional difference in the coupling coordination degree of the eight economic regions vary greatly, and the inter-regional differences between the Northwest Economic Zone and the Middle Yellow River Economic Zone and the Coastal Economic Zone are the main reason for the large inter-regional differences among the eight economic regions; the main source of difference in the coupling coordination degree of energy-economy-carbon emission system in China is the inter-regional differences. Fourth, there is a positive spatial correlation in the coupling coordination degree of energy-economy-carbon emission system; except the σ convergence of the coupling coordination degree of energy-economy-carbon emission system in the Southern Coastal Economic Zone and the Middle Yangtze River Economic Zone, there is no significant σ convergence or β convergence in other economic zones; the coupling coordination degree of energy-economy-carbon emission system in the Middle Yangtze River Economic Zone has the fastest change.

Based on this study, there are several recommendations. First, the coupling coordination degree of the energy-economy-carbon emission system is increasing, and it is important to fully recognise the current efforts made by China to promote high-quality economic development and energy conservation and emission reduction. In the future, the three subsystems of energy, economy and carbon emission will promote each other and co-exist harmoniously, and the prospect of achieving "Carbon Neutrality" and high-quality economic development is bright. At the same time, it is also necessary to face up to the fact that the coupling coordination degree of energy-economy-carbon emission system in China is still at a low level, and to continuously increase the amount of investment in carbon governance. Second, maintain the advantages of the coupling coordination of the energy-economy-carbon emission system in the Eastern Coastal Economic Zone, the Northern Coastal Economic Zone, the Southern Coastal Economic Zone and the Middle Yangtze River Economic Zone, and in particular to give full play to the role of Zhejiang Province, Shanghai, Beijing, Guangdong Province, Fujian Province, Hubei Province, Anhui Province and Jiangxi Province in radiating and driving neighbouring provinces and municipalities; at the same time, focus on the coupling coordination of energy-economy-carbon emissions in the Northeast Economic Zone, the Middle Yellow River Economic Zone and the Northwest Economic Zone, analyze in depth the reasons for thelow level of coupling coordination, and increase financial expenditure in accordance with local conditions to promote the increase in the amount of investment in carbon control. Third, reduce the inter-regional difference among the eight economic zones, especially the inter-regional differences between the Northwest Economic Zone and the Middle Yellow River Economic Zone with coastal economic zones, so as to cut down their destructive effects on the overall coupling coordination of energy-economy-carbon emissions. Four, it is necessary to make full use of the positive spatial correlation of the coupling coordination of energy-economy-carbon emissions, increase the regional correlation and the construction of regional city clusters such as "Yangtze River Delta" and "Pearl River Delta", unswervingly implement the main functional zone system, improve the mechanism of coordinated regional development, give full play to the radiation-driven role of coastal economic zones, especially the Middle Yangtze River Economic Zone, to realize the coupling coordination and balanced regional development of energy-economy-carbon emissions.

Limitations and future research: (1) Regarding the measurement of coupling coordination, this paper only measures the coupling coordination between the three primary indicators of economy, energy and carbon emissions, and does not further calculate the coupling coordination between the detailed indicators, future research can study the coupling coordination relationship between the detailed indicators in depth; (2) This study only includes data from China and not from other countries. The coupling coordination of energy-economy-carbon emission systems in other countries and the comparison between different countries is also a topic worth studying.
